# These boots are made for burnin’: Inferring the position of the corpse and the presence of leather footwears during cremation through isotope (δ^13^C, δ^18^O) and infrared (FTIR) analyses of experimentally burnt skeletal remains

**DOI:** 10.1371/journal.pone.0257199

**Published:** 2021-10-13

**Authors:** Kevin Salesse, Elisavet Stamataki, Ioannis Kontopoulos, Georges Verly, Rica Annaert, Mathieu Boudin, Giacomo Capuzzo, Philippe Claeys, Sarah Dalle, Marta Hlad, Guy de Mulder, Charlotte Sabaux, Amanda Sengeløv, Barbara Veselka, Eugène Warmenbol, Martine Vercauteren, Christophe Snoeck

**Affiliations:** 1 Research Unit: Anthropology and Human Genetics, Department of Biology of Organisms and Ecology, Université Libre de Bruxelles, Brussels, Belgium; 2 UMR 5199: “PACEA–De la Préhistoire à l’Actuel: Culture, Environnement et Anthropologie”, University of Bordeaux, Pessac Cedex, France; 3 Maritime Cultures Research Institute, Department of Art Sciences and Archaeology, Vrije Universiteit Brussel, Brussels, Belgium; 4 Research Unit: Analytical, Environmental and Geo-Chemistry, AMGC-WE-VUB, Vrije Universiteit Brussel, Brussels, Belgium; 5 GLOBE Institute, Section for GeoGenetics, University of Copenhagen, København, Denmark; 6 Faculté des Lettres, Sorbonne Université, Paris, France; 7 Flemish Heritage Agency, Brussels, Belgium; 8 Royal Institute for Cultural Heritage, Brussels, Belgium; 9 Department of Archaeology, Ghent University, Ghent, Belgium; 10 Centre de Recherches en Archéologie et Patrimoine, Department of History, Arts, and Archaeology, Université Libre de Bruxelles, Brussels, Belgium; 11 G-Time Laboratory, Université Libre de Bruxelles, Brussels, Belgium; University of Padova: Universita degli Studi di Padova, ITALY

## Abstract

Cremation is a complex mortuary practice, involving a number of activities of the living towards the dead before, during, and after the destruction of the bodily soft tissues by fire. The limiting information concerning these behavioral patterns obtained from the pyre remains and/or cremation deposits prevents the reconstruction of the handling of the corpse during the burning process. This pioneering study tries to determine the initial positioning of the corpse in the pyre and assess whether the deceased was wearing closed leather shoes during cremation through isotopic (δ^13^C, δ^18^O) and infrared (ATR-FTIR) analyses of experimentally burnt pig remains, used as a proxy for humans. The results obtained show that both the position of feet on or within the pyre and the presence of footwears may moderately-to-highly influence the oxygen isotope ratios of bone apatite carbonates and the cyanamide content of calcined bone in certain situations. By forming a protective layer, shoes appear to temporarily delay the burning of the underlying pig tissues and to increase the heat-shielding effect of the soft tissues protecting the bone mineral fraction. In such case, bioapatite bone carbonates exchange oxygen with a relatively more ^18^O-depleted atmosphere (due to the influence of lignin-derived oxygen rather than cellulose-derived oxygen), resulting in more pronounced decrease in the δ^18^O_carb_ values during burning of the shoed feet vs. unshoed feet. The shift observed here was as high as 2.5‰. A concomitant isotopic effect of the initial location of the feet in the pyres was also observed, resulting in a top-to-bottom decrease difference in the δ^18^O_carb_ values of shoed feet of about 1.4‰ between each deposition level tested. Finally, the presence of cyanamide (CN/P ≥ 0.02) seems to be indicative of closed footwear since the latter creates favorable conditions for its incorporation into bone apatite.

## Introduction

Cremation is a complex process that has been performed in a wide range of geographic and cultural contexts from prehistory to present day [1–8], involving a chain of activities of the living towards the dead before, during, and after cremation [9, 10]. The investigation of the post-cremation history of burnt skeletal remains is particularly complicated due to their fragmentary and often incomplete nature [11–14], but reconstructing the handling of the corpse during the burning process probably constitutes an even more challenging venture. The ephemerality of the moment, the taphonomic vulnerability of the pyre locations [15–17], the choices made by the funerary ritual attendants, and the unpredictable contingencies inherent to the burning process (e.g. pyre collapse) are all variables that make each cremation a unique event [9, 18–20]. Yet, to better understand the cremation processes and, ultimately, the funerary ritual determining how the body of the deceased was managed during burning is vital (see [21] or [22] for archaeological examples; see [23] for ethnographical cases).

In open-air cremations, the manipulation of the corpse during the burning process is conditioned, mainly but not only, by its initial positioning in the pyre, and by the presence of clothing (shroud, garments, etc.), both of which can affect the efficiency of combustion in a variety of ways. Although it is commonly suggested that the deceased is placed on top of the pyre to be visible during the burning phase (e.g. [16]), other arrangements include placing the corpse under or in the heart of the pyre structure [24–26]. The deceased may have been previously clothed, placed in a shroud or left naked [24, 27]. When exposed to fire, clothing–typically made of vegetal fabrics or animal leathers [25]–is swiftly consumed, making its presence difficult to establish, unless metalwork, such as ornaments (e.g. belt buckles, brooches), reinforcements (e.g. hobnails, toe tips) or others accessories (e.g. pins, buttons) is recovered from the pyre remains [25, 26]. The handling of the corpse, its positioning and dressing, may differ from one individual and/or population to another depending on many factors, such as the biological and social status of the deceased [28–32], the religious beliefs and cultural practices of the participants in the funerary event [24, 32–34], and/or the expertise of the cremation operators [35]. Identifying the positioning and dressing of the corpse improves understanding of past societies that practiced cremation as a funeral ritual.

Recent work has highlighted the high potential of calcined skeletal remains to isotopically investigate cremation practices (see [36] and references therein). While all organic matter has been destroyed by the high temperatures reached during the burning process, the inorganic fraction (often called bone apatite or bioapatite) still contains a certain amount of carbon and oxygen under the form of carbonates. Lanting and colleagues [37, 38] showed that while it was possible to obtain reliable radiocarbon (^14^C) ages from the carbonate fraction of calcined bones in specific archaeological contexts, the stable carbon isotope compositions (δ^13^C) of these bones had been significantly altered. It was proposed that three parallel mechanisms could explain these alterations. First, carbon exchanges occur between the bone apatite carbonates and the surrounding cremation atmosphere. Second, carbon from soft tissues and bone collagen admixes to that of the bone apatite carbonates during heating. Third, most of the structural bone apatite carbonates are lost during the combustion process, and this loss is likely associated with time/temperature-dependent fractionation [39–50]. Experimental works have shown that fuel carbon replaces up to 95% of the biogenic bioapatite carbon as a result of these mechanisms [42, 47, 49].

Less consideration has been given to the stable oxygen isotope composition of calcined bone apatite carbonates (δ^18^O_carb_). While a temperature-related change in the δ^18^O_carb_ values of calcined bones was consistently recognized, the explanatory model behind this shift remains not yet fully understood [48, 50–53]. Indeed, in a cremation environment, many possible sources of oxygen can ultimately influence the bone δ^18^O_carb_ signals (e.g. O_2_, CO, CO_2_, H_2_O, NO_x_ from the ambient air, fuel or human/animal soft tissues and bone collagen). Oxygen isotope exchanges between bone apatite carbonates and CO_2_ from the combustion atmosphere–and particularly from fuel–could have led to the observed ^18^O depletion in calcined bones [50]. Temperature-induced isotopic fractionation associated with the loss of structural carbonates during the combustion process may also be at the origin of the decrease in the δ^18^O_carb_ values of calcined bones [50, 53]. Even if the δ^13^C_carb_ and δ^18^O_carb_ proxies can no longer be used to infer life traits (i.e. diet and mobility) of the deceased in cremation contexts, they can be used as semi-independent lines of evidence to better characterize cremation conditions [50, 54, 55].

Fourier transform infrared (FTIR) indices offer the possibility to assess the extrinsic and/or intrinsic factors at the origin of the cremation-induced transformations [52, 56–60]. During cremation, bone is physically and chemically altered. These alterations occur in a generally orderly manner. As the temperature rises, the thermal shielding effect of the organic matter protecting the inorganic fraction diminishes and it is probably only after this loss that the carbonates are completely exposed to heat, and thus gradually lost [61–64]. The CO_2_ emitted during the combustion of bone initially comes from the organic matter, and in a second stage from structural carbonates, with a transition period between the two [65]. As a consequence of heating, alterations in bone crystallinity occur, with larger crystals and higher degrees of crystallinity, making burnt bone, and in particular calcined bone, more resistant to post-burial alterations [57, 60]. Throughout its rearrangement, bone apatite can incorporate new chemical species such as cyanamide. Cyanamide molecules (H_2_CN_2_) form in the presence of ammonia and carbon, and ${\mathrm{CN}}_{2}^{2-}$ substitute for hydroxyl (OH^−^) groups or type A carbonates in the apatite lattice [66–69]. The origin of ammonia is still uncertain; however, it could be a byproduct of the combustion of organic matter (e.g. body tissues) or fuel (e.g. coal) [45, 47]. A greater occurrence of cyanamide is expected to be found when combustion occurs under reducing conditions (i.e. low oxygen availability) [43, 50, 57, 70]. Therefore, the cyanamide content constitutes an useful indicator for identifying specific cremation settings.

This study aims to better understand past cremation practices and related funerary rituals by identifying the initial positioning of the corpse deposited on/in a pyre and assess whether the deceased was wearing closed leather shoes during cremation through isotopic (δ^13^C_carb_ and δ^18^O_carb_) and infrared (ATR-FTIR) analyses of experimentally burnt pig remains. Leather footwear is postulated to be the most fire-resistant type of clothing and foot bones encased in a closed type of leather shoe may experience heating conditions characteristic of confined-space cremation (low availability of oxygen, poor ventilation, etc.), which could lead to distinct bone apatite chemical and/or structural variations. The effects of the deposition levels of the feet (i.e. bottom, middle, or top of pyre) are also investigated to establish whether their initial position affects their chemical and structural characteristics due to different heating conditions.

## Materials and methods

### Setting up and running of experimental cremations

The experimental cremations were conducted in Wallonia (Belgium) during two different sessions (October 2018 and July 2019), the first in a clearing partially sheltered from the wind and the second in a disused quarry more exposed to the wind. Four fires were conducted per session.

European beech (*Fagus sylvatica*) wood was selected to build all eight experimental pyres (see Section 1 in S1 Appendix for the description of the pyre architecture). The firewood was cut from dried logs (n ≃ 12) originating from a small plot of land located in the French Ardennes (Houdain Lez Bavay, France). Vegetable tanned cow and goat leathers were purchased (Steffisburg, Switzerland) to make the experimental footwear. With a thickness of 3 mm and 1.5 mm respectively, these leathers were used to test the fire resistance of two different types of shoes. Seven shoes were created using the cowhide and only one with the goatskin (see Section 1 in S1 Appendix for details about the leather manufacturing process and the footwear making). Domestic pigs (*Sus scrofa domesticus*) were used as human substitutes since carcasses or body parts of such animals have long been involved in experimental cremations [71–74]. Feet of nine pigs raised in the same farm were bought from a local butcher (Anderlecht/Sint-Lievens-Houtem, Belgium).

In each experimental outdoor cremation, one unshoed foot and one shoed foot from a single animal were placed at the same level in the pyre structure and as far apart as possible from each other (ca. 40 cm). For each experimental session, pig feet were placed on top (x 1), in the middle (x 2) or at the bottom (x 1) of the pyres. Dry farmyard manure pellets (≃ 5 kg) were added to one of the pyres (pyre 8) to create an ammonia-rich combustion atmosphere. The goatskin was used in only one case (pyre 3). Feet from the ninth pig were burned one by one in a muffle furnace (Thermo Heraeus M110) at 900°C for about 5h and allowed to cool down overnight.

**Fig 1:**
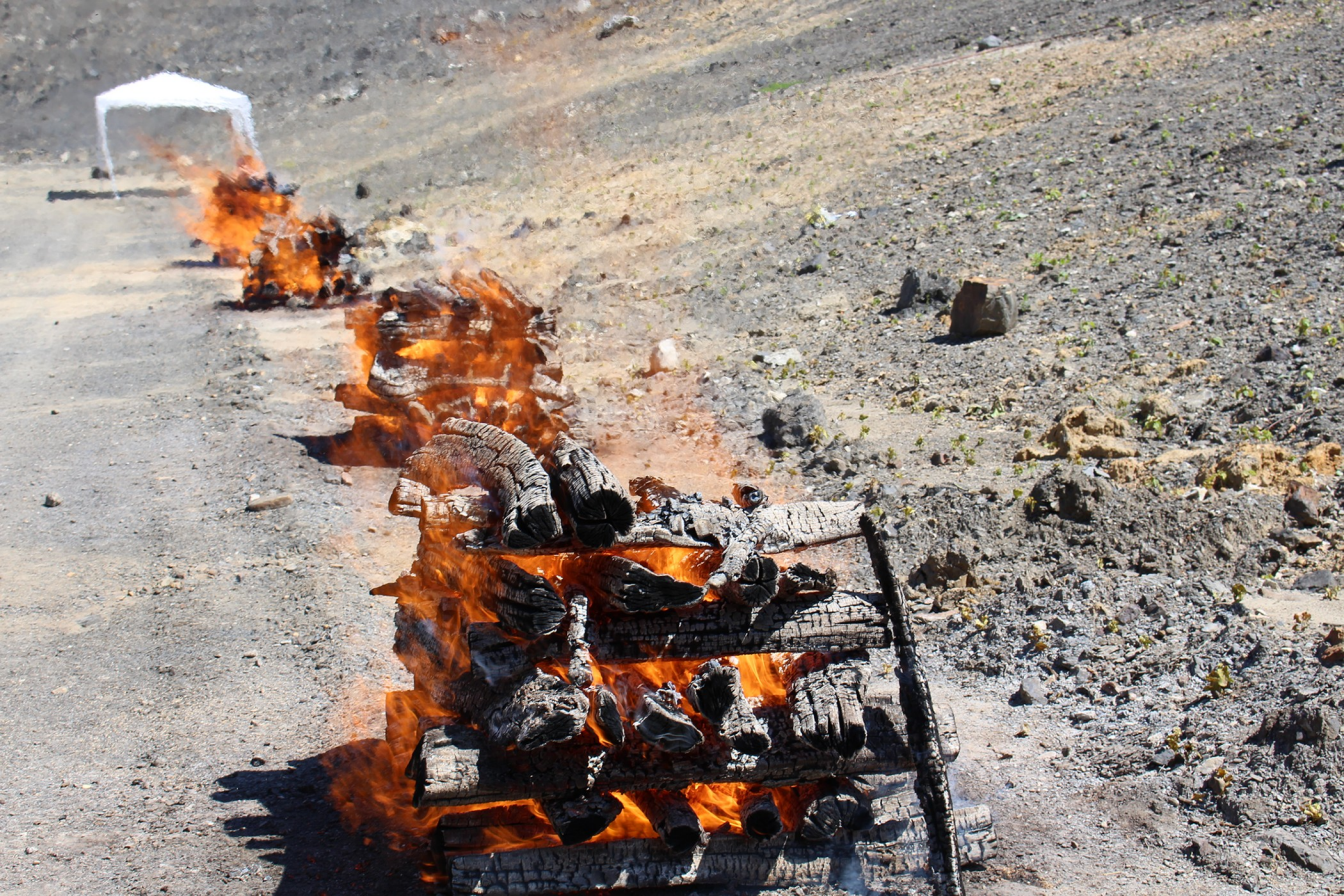
Active burning of experimentally constructed pyres. Credits: Ahmad Alsaadi.

On average, the pyres were reduced to ashes after 1–1.5h. All the pyres reached a temperature of 900°C and some went above 1000°C for a short period of time (S2.1 Table in S2 Appendix). There was no human intervention after the fire was lit (Fig 1) and no additional fuel was loaded into the fires during the cremation process. All structures, apart from pyre 7, collapsed on themselves, retaining the animal skeletal remains within. Pyre 7 toppled chaotically to the side, causing the shoed foot to fall out of the pyre. In all cases, the burning process lasted long enough to destroy the organic matter (leathers, pig soft tissues and bone collagen) and calcine most of the recovered foot bones. In the first session, the pyre remains were rapidly cooled down due to heavy rains during the night, while those burned in the second session were allowed to cool down naturally overnight. Bones were recovered on the top and within the ash piles on the day following the cremation experiments.

### Pre-treatments and stable isotope analysis of materials used in the experiments

The metacarpals/metatarsals from unburnt pig feet were isolated by dissection, and the periosteum was removed with a scalpel. Bones were cleaned using a tungsten carbide drill bit to retain only the cortical part. Bones were then ground into powder using a countertop blender. Particles ranging between 0.3 and 0.7 mm were collected for collagen extraction, and those less than 0.3 mm were used for carbon and oxygen isotope analyses of bioapatite carbonates. Prior to the purification procedures, bone powder samples were defatted according to the protocol developed by Kates [75] and tested by Liden et al. [76], as presented in Salesse et al. [77]. Bone collagen was extracted following the non-ultrafiltered procedure proposed by Brock et al. [78] while unburnt bone carbonate samples were prepared following a revised version of the protocol of Balasse et al. [79]. With regards to the calcined material, the outer layers of the bone fragments were scratched with a scalpel. The samples were prepared according to the procedure described by Snoeck et al. [80]. Between 4 and 7 different (calcined) bones per foot were sampled (amount: ca. 500 mg per sampled bone). Wood shavings, animal hide cuts and soft tissues of the pig (skin, muscle, and tendon) were sampled using a scalpel. They received no further treatment prior to isotopic analysis. All these pre-treatments were carried out at the AMGC Research Unit of the *Vrije Universiteit Brussel* (VUB, Brussels, Belgium). More information about the pre-treatments carried out is provided in Section 2 in S1 Appendix.

Carbon and nitrogen abundances and isotope compositions for organic samples (amount: ≈ 500 μg, in tin capsule) were measured in duplicate on different aliquots using a Eurovector Elemental Analyzer (EA) coupled with a Nu Perspective Isotope Ratio Mass Spectrometer (IRMS) at the AMGC Research Unit (S3.1, S3.2 and S4.1 Tables in S3 and S4 Appendices). The δ^13^C_organic_ and δ^15^N_organic_ values are reported as per mil (‰) difference relative to VPDB and AIR, respectively. Standards (IA-R041: δ^13^C = -23.3‰ and δ^15^N = -5.6‰; IAEA-C-6: δ^13^C = -10.8‰; IAEA-CH-7: δ^13^C = -32.2‰; IAEA-N-1: δ^15^N = +0.4‰; IAEA-N-2: δ^15^N = +20.3‰) were used for data correction. All samples and standards were analyzed randomly except IA-R041 that was used for instrumental drift correction. Analytical errors were better than ± 0.2‰ (1SD) for both δ^13^C and δ^15^N. Quality criteria for bone collagen–extraction yields (%Col), carbon and nitrogen contents (%C and %N), and atomic C:N ratios–are presented in S4.1 Table in S4 Appendix.

Carbon and oxygen isotope compositions for unburnt and calcined bone samples (amount: ≈ 2 mg) were measured in duplicate on different aliquots via a NuCarb carbonate preparation device interfaced with a Nu Perspective IRMS (first batch) or via the NuCarb/IRMS system including a Nu GasPrep automatic gas sampler (second batch) at the AMGC Research Unit (S4.1 and S4.2 Tables in S4 Appendix). The results are reported as per mil (‰) deviation from VPDB reference standard scale. Two internal standards were used (ENF: δ^13^C = -9.8‰ and δ^18^O = -5.4‰; CBA: δ^13^C = -14.8‰ and δ^18^O = -10.8‰ –see de Winter et al. 2016), as well as Iso-Analytical IA-R022 (δ^13^C = -28.6‰ and δ^18^O = -22.7‰), and international standards IAEA-603 (δ^13^C = 2.5‰ and δ^18^O = -2.3‰), and IAEA-CO-8 (δ^13^C = -5.8‰ and δ^18^O = -22.7‰). Over the course of all analyses, the analytical precision was better than ± 0.25‰ (1SD) for both δ^13^C_carb_ and δ^18^O_carb_ based on repeated measurements of CBA (n = 26). Δ is used to express isotopic differences between unshoed and shoed feet of the same pig (e.g. Δ^13^C_shoed-unshoed_ or Δ^18^O_shoed-unshoed_).

### Preparation and ATR-FTIR analysis of burnt bones

Only the pig bones from the second burning experiments were studied using infrared analyses. Sample preparation and infrared analysis of calcined bone were carried out according to Kontopoulos et al. [81] (see Section 2 in S1 Appendix for specifics). Bioapatite changes were assessed through FTIR spectroscopy using attenuated total reflection (ATR). Between 5 and 7 different bones per foot were investigated. ATR-FTIR measurements on bone samples (amount: 2 to 3 mg per sampled bone) were performed in triplicate under vacuum on different aliquots using a Bruker Vertex 70v FTIR spectrometer (range: 4000–400 cm^-1^; No. of scans: 64; resolution: 4 cm^-1^; mode: absorbance) at the AMGC Research Unit. A background measurement was run before each sample analysis to remove the background signal. After each measurement, the crystal plate and the anvil of the pressure applicator were cleaned using a dry Kimtech^TM^ precision wipe. The Bruker OPUS software (v. 7.5) was used to determine the infrared indices (see Section 2 in S1 Appendix for the list of measured indices). Differences in infrared proxies between feet are expressed by using Δ (e.g. ΔCN/P_shoed-unshoed_; see S5.2 Table in S5 Appendix).

The Bruker Vertex 70v is a high performance spectrometer equipped with an evacuable optics bench, a RockSolid^TM^ interferometer and DigiTect^TM^ 24-bit ADC detectors. These features ensure improved sensitivity and spectral resolution as well as lower electronic noise and atmospheric interferences compared to conventional FTIR spectrometers. Because the detection threshold for cyanamide has been established on a conventional FTIR spectrometer operating at standard atmospheric pressure [57], an instrument intercomparison was performed for testing comparability of the CN/P values between studies. Samples initially measured by Snoeck et al. [57] using an Agilent Technologies Cary 640 (no vacuum) were rerun using the Bruker Vertex 70v (under vacuum) from the AMGC Research Unit.

## Results and discussion

### δ^13^C_carb_ signals: The fuel effect

The δ^13^C_carb_ values of pig foot bones shift drastically during the cremation process. While the unburnt bones have δ^13^C_carb_ values ranging from -15.4 to -14.8‰ (S4.1 Table in S4 Appendix), the calcined bones (from all burning experiments) have δ^13^C_carb_ values ranging from -29.8‰ to -17.8‰ (S4.2 Table in S4 Appendix). On average, the δ^13^C_carb_ values of the unburnt bones (mean = -15.1 ± 0.2‰, 1SD) differ by -10.2‰ from the burnt bones (mean = -25.3 ± 1.9‰, 1SD).

**Fig 2:**
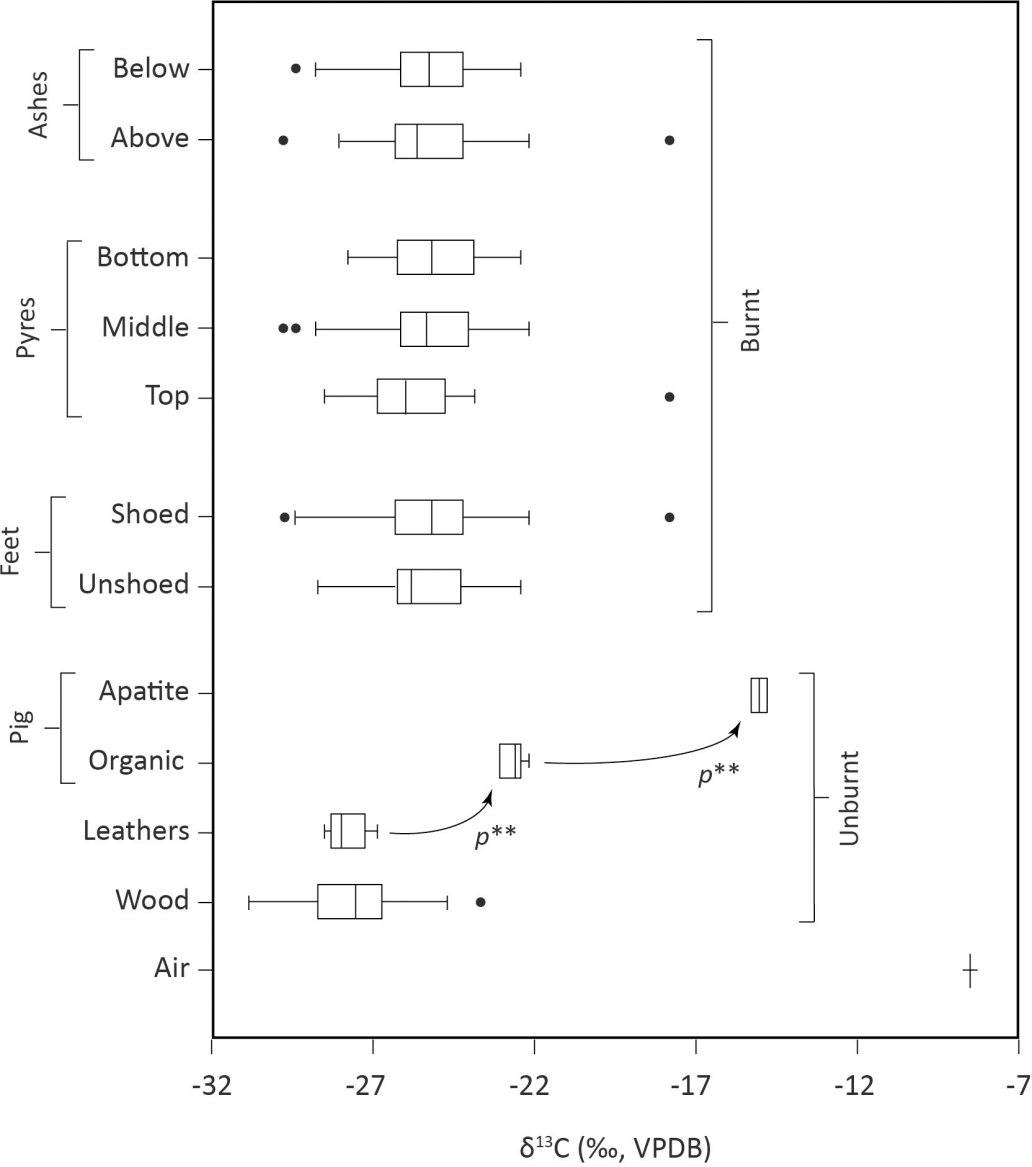
Boxplots presenting the distribution of the δ^13^C values measured on the wood, leathers and pig tissues. p** represents statistically significant differences (based on Mann-Whitney U tests).

Heating experiments carried out in a fuel-free muffle furnace (pig 9) show that the δ^13^C_carb_ values of calcined bones (mean_[unshoed]_ = -20.6 ± 0.5‰, 1SD; mean_[shoed]_ = -24.6 ± 0.9‰, 1SD; S4.2 Table in S4 Appendix) tend to decrease towards the δ^13^C values of the pig skin, muscle, tendon and bone collagen (mean = -23.1 ± 0.8‰, 1SD; S4.1 Table in S4 Appendix) and/or the leathers (mean = -27.9 ± 0.6‰, 1SD; Section 3 in S1 Appendix and S3.1 Table in S3 Appendix) (Fig 2). This suggests that part of the carbon exchanges between bone apatite carbonates and combustion gases–especially CO2 –occurred during the burning of organic matter, including leathers. However, these bones exhibit less negative δ^13^C_carb_ values than those cremated in outdoor experiments (pigs 1 to 8; mean = -25.4 ± 1.8‰, 1SD; S4.2 Table in S4 Appendix).

When cremated on pyres, the unshoed feet yield δ^13^C_carb_ values ranging from -28.8 to -22.5‰ (mean = -25.5 ± 1.5‰, 1SD) while the shoed feet have δ^13^C_carb_ values ranging from -29.8 to -17.8‰ (mean = -25.4 ± 2.1‰, 1SD) (Fig 2; Table 1 and S4.2 Table in S4 Appendix). There is no significant difference in δ^13^C_carb_ values between these two groups of feet (Mann-Whitney (MW) test, *p* = 0.74; S6.2 Table in S6 Appendix). This absence of difference is also observed within each pyre and between pyres (MW tests, p ≥ 0.06; S6.3 Table in S6 Appendix) (Fig 2). Still, the larger variability in δ^13^C_carb_ values seen in shoed feet (± 2.1‰, 1SD) compared to unshoed feet (± 1.5‰, 1SD) might be linked to the presence of more carbon sources and/or more variable burning conditions for the shoed feet because of the presence of footwear. The δ^13^C_carb_ values of bones recovered above or below the ashes are similar (MW test, *p* = 0.81; S6.4 Table in S6 Appendix) (Fig 2). Therefore, neither the presence of footwear nor the initial position of the feet in the pyres nor the final location of the bones in the ashes seem to visibly affect the δ^13^C_carb_ values of calcined bones.

**Table 1:** Mean δ^13^C_carb_ and δ^18^O_carb_ values and discrepancies between feet of experimentally cremated pigs.

Sample	Position	SHOED (‰ –VPDB)					UNSHOED (‰ –VPDB)					DIFFERENCES (‰ –VPDB)			
		n	δ^13^C_carb_	SD	δ^18^O_carb_	SD	n	δ^13^C_carb_	SD	δ^18^O_carb_	SD	Δ^13^C_shoed-unshoed_	SD	Δ^18^O_shoed-unshoed_	SD
**Pig 1 / Pyre 1**	Top	4	-26.6	1.9	-14.8	1.5	5	-26.0	1.5	-15.2	1.8	-0.6	0.9	0.4	0.8
**Pig 2/ Pyre 2**	Middle	5	-26.3	3.3	-16.5	1.4	5	-26.6	0.8	-15.1	0.5	0.3	0.9	-1.4	0.6
**Pig 3 / Pyre 3** *	Middle	4	-24.9	2.0	-15.3	0.6	5	-25.3	1.9	-14.3	1.4	0.4	0.9	-1.0	0.7
**Pig 4 / Pyre 4**	Bottom	5	-26.5	1.2	-18.3	1.7	4	-24.0	2.5	-15.9	1.7	-2.5	0.9	-2.4	0.7
**Pig 5 / Pyre 5**	Bottom	5	-25.5	0.9	-18.8	0.4	5	-24.8	1.1	-16.3	0.6	-0.7	0.6	-2.5	0.5
**Pig 6 / Pyre 6**	Middle	6	-24.7	1.1	-17.1	1.0	6	-25.8	0.7	-16.7	1.2	1.1	0.5	-0.4	0.6
**Pig 7 / Pyre 7**	Top	7	-24.7	3.3	-16.2	1.1	6	-25.9	0.8	-15.2	0.8	1.2	0.8	-1.0	0.5
**Pig 8 / Pyre 8** ^**ⴕ**^	Middle	6	-24.5	0.9	-15.4	1.0	5	-25.1	2.2	-15.8	1.2	0.6	0.8	0.4	0.6
**Pig 9 / Furnace**	900°C / 5h	2	-24.6	0.9	-14.4	0.3	2	-20.6	0.5	-15.7	0.3	-4.0	0.8	1.3	0.5

Full data available in S4.2 Table in S4 Appendix.

*Pyre where the pig foot was placed in goatskin instead of cow leather.

^ⴕ^Dry farmyard manure pellets were added to this pyre.

This is most likely because wood is present in much larger amounts and, when burned, releases much larger quantities of CO_2_ compared to any other material cremated simultaneously. Wood-derived carbon thus not only participates predominantly in the various chemical exchanges that occur in the cremation environment, but also largely influences the isotopic signature of the CO_2_ present in the cremation atmosphere. Still, in none of the experiments carried out did the δ^13^C_carb_ values of calcined bones perfectly match the δ^13^C_org_ values of the pig soft tissues, the pig bone collagen, the leathers, or the firewood. This observation is consistent with the results of previous work showing that most but not all the carbon in the carbonate fraction of calcined bones is replaced by carbon from the fuel [42, 47, 49].

### δ^18^O_carb_ signals: The local heating conditions

**Fig 3:**
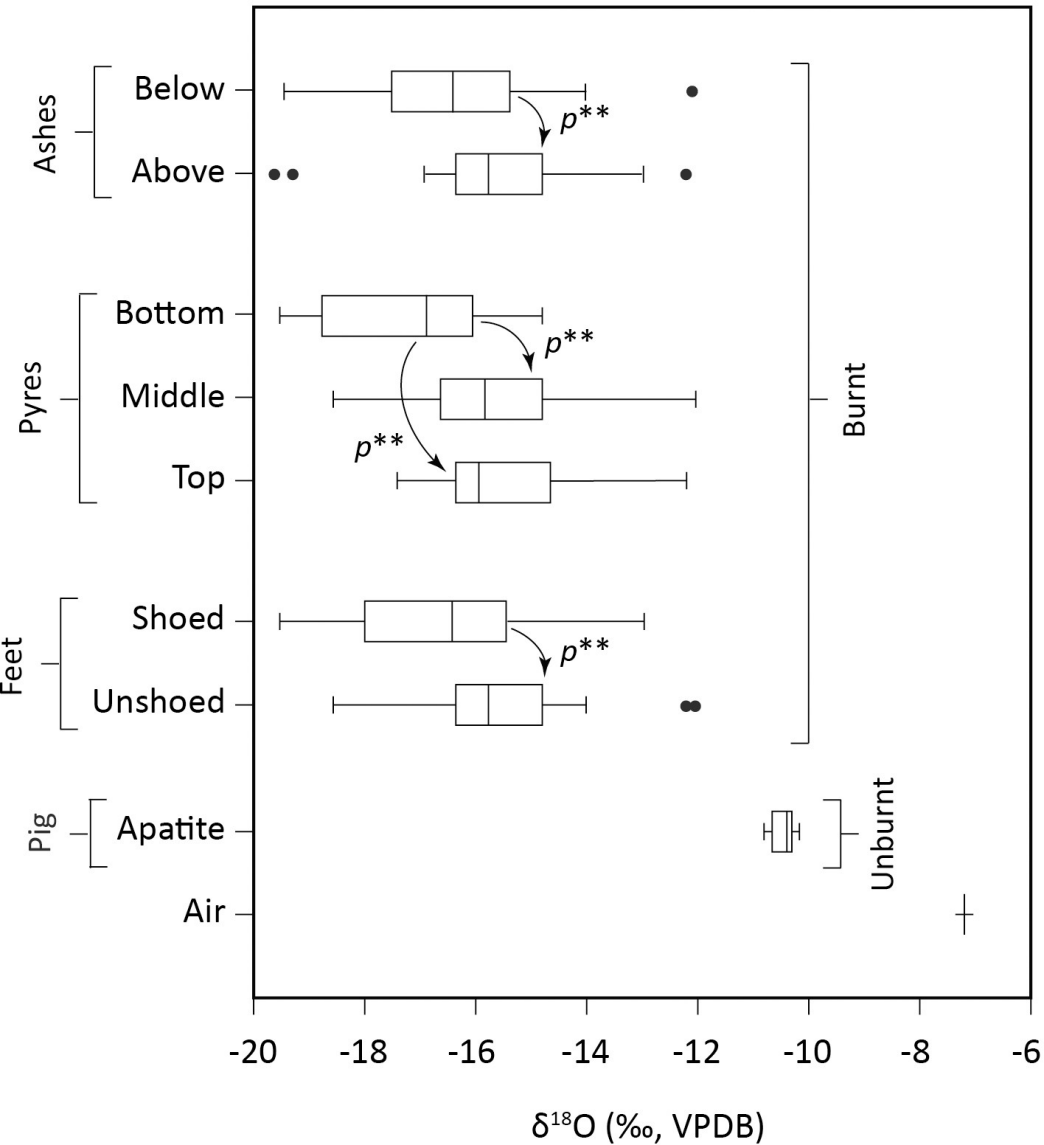
Boxplots presenting the distribution of the δ^18^O values measured on the wood, leathers and pig tissues. p** represents statistically significant differences (based on Mann-Whitney U tests).

During the burning process, the δ^18^O_carb_ values of pig foot bones drop similarly to the δ^13^C_carb_ values (Fig 3). While the raw bones have δ^18^O_carb_ values ranging from -10.8 to -10.2‰, (S4.1 Table in S4 Appendix), the calcined bones (from all burning experiments) have δ^18^O_carb_ values ranging from -19.6‰ to -12.1‰ (S4.2 Table in S4 Appendix). On average, the δ^18^O_carb_ values of the unburnt bones (mean = -10.4 ± 0.2‰, 1SD) differ by -5.6‰ from the burnt bones (mean = -16 ± 1.6‰, 1SD).

The unshoed and shoed feet from the laboratory burning experiments have δ^18^O_carb_ values that differ by 1.3‰ on average. Such a pattern is also found in most of the outdoor burnings (Tables 1 and S3.2 Table in S3 Appendix). When cremated on pyres, the unshoed and shoed feet have δ^18^O_carb_ values ranging from -18.6 to -12.1‰ (mean = -15.6 ± 1.3‰, 1SD) and from -19.6 to -13‰ (mean = -16.6 ± 1.7‰, 1SD), respectively (Fig 3; Tables 1 and S3.2 Table in S3 Appendix). The difference in δ^18^O_carb_ values between the two groups of feet is statistically significant (MW test, *p* = 0.01; S6.5 Table in S6 Appendix) (Fig 3). If this difference is examined per pyre, the outcome is less straightforward. There is no difference in δ^18^O_carb_ values between the feet placed on top or in the middle of the pyres (MW tests, *p* ≥ 0.18; S6.6 Table in S6 Appendix) (Fig 3). In contrast, when deposited at the bottom of the structure, the δ^18^O_carb_ values of the unshoed feet statistically significantly differ from the shoed feet by 2.5‰ (MW test, *p* < 0.01; S6.6 Table in S6 Appendix) (Fig 3). Overall, the burnt shoed feet are more depleted in ^18^O than the burnt unshoed feet, demonstrating a certain isotopic effect of wearing closed leather footwear during cremation.

**Fig 4:**
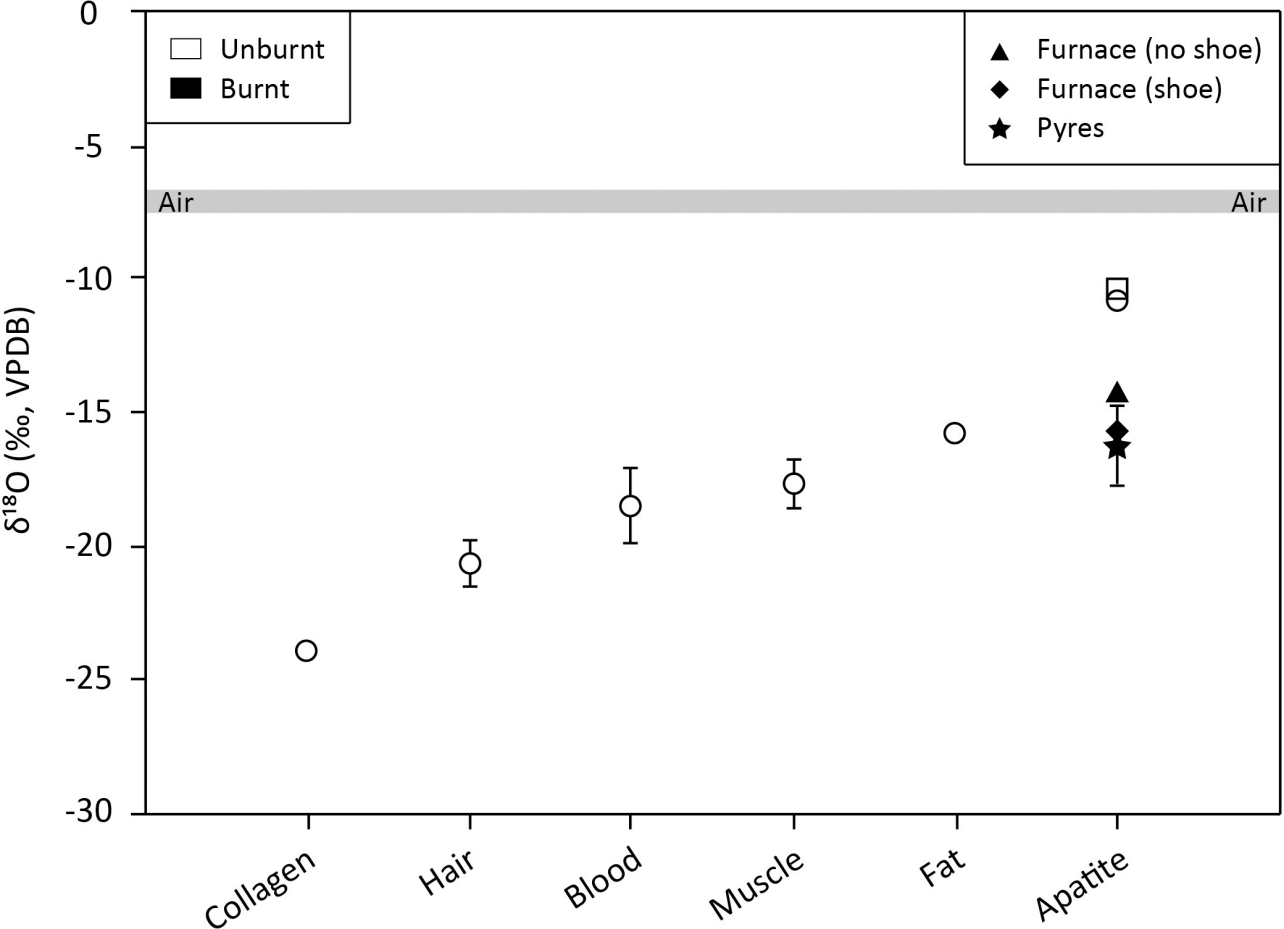
δ^18^O values of pig tissues from this study (square, triangle, diamond, star) compared to the dataset from Tuross et al. [82] and Warinner and Tuross [83] (circle).

By forming a temporary protective layer around the foot, the shoe creates conditions that prolong the pyrolysis of the underlying pig tissues, while delaying the ignition and subsequent burning processes of the latter. Inside the shoe, pyrolysis-derived volatiles, gases and residues would accumulate, setting up a specific local atmosphere, with δ^18^O values distinct from those on the outside. The organic δ^18^O values (hides/skins used for footwear) are expected to be lower than mineral δ^18^O values. For pigs with similar δ^18^O_carb_ values to those obtained in this study (-10/-11‰), Tuross et al. [82] as well as Warinner and Tuross [83] found that bone collagen (-23.9 ± 0.2‰), hair (-20.7 ± 0.9‰), blood (-18.5 ± 1.4‰), muscle (-17.7 ± 1‰) and fat (-15.8 ± 0.5‰), were depleted in ^18^O compared to unburnt bone apatite (-10.9 ± 0.4‰) (Fig 4). Such differences between tissues are also recorded for other domesticated herbivores (e.g. [84, 85]). Based on the environment in which the cattle and goats used for leather production were reared, the hides/skins are expected to have δ^18^O values in the range of those of the pig organic tissues.

However, oxygen exchanges between the inner shoe atmosphere and the bone apatite carbonates are thought to be minimal. If structural rearrangements or chemical changes in apatite have been reported at low temperatures, this generally concerns synthetic carbonated hydroxyapatites (e.g. AB-CO_3_-Aps) or skeletal tissues containing a small quantity of organic matter (e.g. tooth enamel) [86, 87]. In bones, soft tissues and collagen matrix shield the inorganic fraction, delaying carbonates exposure to heat and consequently restricting the oxygen exchanges with the surrounding atmosphere [61–64]. Meanwhile in the pyre, wood is consumed under the action of fire which is conditioned by the thermal degradation properties of the wood compounds: i.e. holocellulose and lignin. Holocellulose starts to burn first, followed by lignin. Initially largely influenced by holocellulose, the combustion-derived oxygen pool is then mainly influenced by lignin as burning progresses [88]. These compounds contain both very distinct oxygen isotopic compositions, with (holo-)cellulose being significantly enriched in ^18^O compared to lignin (up to 11‰) [89–92]. The bones from the shoed foot are apparently exposed to fire at a more advanced stage of burning and exchange oxygen with a relatively more ^18^O-depleted atmosphere than bones from the unshoed foot; the leather shoe acting as an additional protective organic layer to the inorganic fraction of the bones.

**Fig 5:**
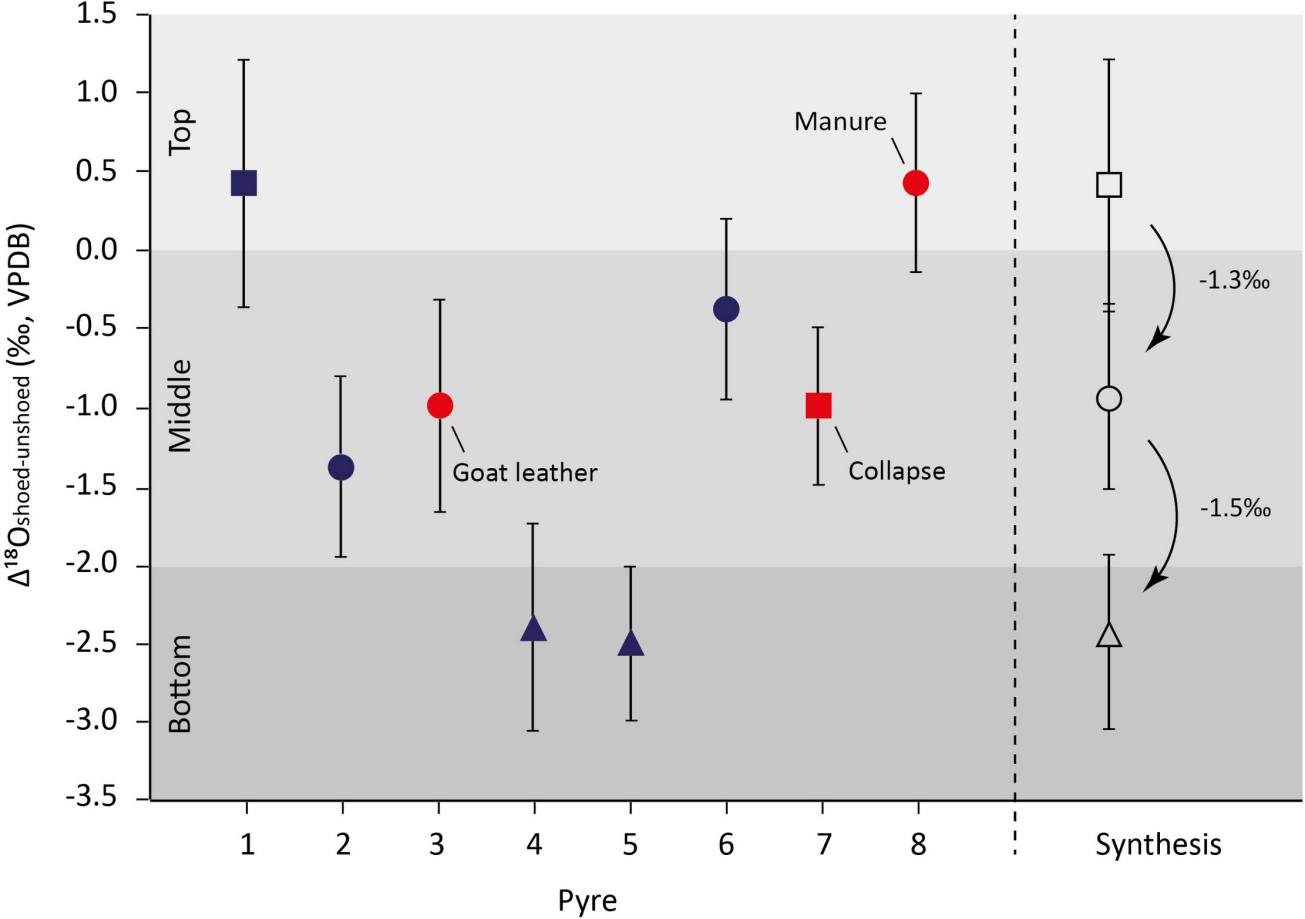
Δ^18^O_shoed-unshoed_ values (± 1SD) of the pigs burned in the experimental cremations. Squares refer to top-position pyres, circles for middle-position pyres, and triangles for bottom-position pyres. The synthesis excludes pyre 7 and 8.

Regardless of footwear, feet put on top or in the middle of the pyres present δ^18^O_carb_ values that statistically significantly differ from those of feet laid out at the bottom of the structure (Δ^18^O_top/middle-bottom_ = 1,7‰; MW test, *p* < 0.01; S6.7 Table in S6 Appendix). Feet deposited at the bottom of pyres exhibit the lowest mean Δ^18^O_shoed-unshoed_ values (below -2.4‰; pyres 4 and 5), while those placed on top present the highest mean Δ^18^O_shoed-unshoed_ value (+0.4‰; pyre 1) (Fig 5; Table 1). The observed decrease in Δ^18^O_shoed-unshoed_ values from the top to the bottom of the pyres suggests that local variations in heating conditions occur. The fires of open-air cremations burn heterogeneously and samples are exposed to varying temperatures and changing atmospheres [93]. Thermal variations could have been larger and exposure times longer at the bottom than on top of the pyres, resulting in distinct exchanges and/or isotope fractionations of oxygen. In addition, the oxygen availability probably changed drastically within the wooden structures. The oxygen supply, the accumulation and removal of combustion by-products, and the interaction with the various gases were probably different in the heart or deep down in the pyres than closer to the surface. Moreover, the diffusion of oxygen could have been more complex or slower inside the pyre than closer to the surface, resulting in different isotopic fractionations and/or exchanges.

Furthermore, the shoes appear to have been subjected to different physical constraints depending on their position in the pyres. The shoes within the pyres stayed closed longer, probably because of the pressure from the firewood, while the shoe placed on top of pyre 1 opened quickly, exposing the foot to the same burning regime than the unshoed foot. This would explain the small but positive difference observed between shoed and unshoed feet recorded for pyre 1 (Δ^18^O_shoed-unshoed_ = +0.4‰). It can also be observed that the mean Δ^18^O_shoed-unshoed_ values decrease by -1.4‰ in average between each position, from top to bottom (Fig 5). This clearly evidences a concomitant influence of the initial position of the feet in the pyres and the wearing of footwear. It also highlights that, regardless the type of leather used, a comparable isotopic effect is noted for the shoed feet placed in the middle of the pyres.

Comparing the Δ^18^O_shoed-unshoed_ results obtained in each burning session, they are strikingly similar, especially for the bones burned at the bottom of the pyre (pyres 4 and 5 –Fig 3; Table 1) suggesting that, at the bottom of a pyre, conditions are quite similar regardless of the location of the pyre within the landscape (enclosed area in session 1 vs open hill in session 2). The bones burned in the middle position also show similar Δ^18^O_shoed-unshoed_ values but the slight difference between pyre 2 (cow leather) and 3 (goat leather) could be explained by the difference in thickness between the two leathers, the latter being thinner and thus burning away faster. The difference between pyres 2 and 6, where pyre 6 has a slightly less negative mean Δ^18^O_shoed-unshoed_ value (-1.4 ‰ and -0.4‰ respectively) could be the results of a difference in openness of the cremation area where an open area could create a more homogeneous combustion atmosphere in the middle area of the pyre.

The results of pyres 7 and 8 further show the impact of fuel and position on the pyre on the oxygen isotope results. While the unshoed foot from pyre 7 has an identical mean δ^18^O_carb_ value to that of pyre 1 (top), the shoed foot from pyre 7 presents a similar mean δ^18^O_carb_ value than those of the shoed feet from pyres 2, 3 and 6 (middle). This is most likely related to the fact that pyre 7 toppled chaotically, subjecting the shoed foot to a different burning regime. When the pyre collapsed, a few pieces of burning wood covered the shoed foot, recreating to some extent an atmosphere comparable to that experienced by the feet placed in the middle of the pyres.

With regards to pyre 8, it can be observed that the shoed foot has a slightly higher mean δ^18^O_carb_ value than the unshoed foot. Manure has a similar decomposition pattern to lignin [94], and, in general, fertilizers appear to have relatively high δ^18^O values [95]. Assuming that the ^18^O-enriched manure could mitigate the influence of the ^18^O-depleted lignin and create a more homogenous combustion atmosphere throughout the cremation process in comparison to the other pyres where cellulose is expected to burn away faster than lignin (i.e. creating a change in combustion atmosphere through time), the shoed and unshoed feet from pyre 8 could have been burned in a more comparable atmosphere.

Whether they were initially encased in shoes or not, the δ^18^O_carb_ values of bones (from all pyres) recovered below the ashes differ from those of bones retrieved from above the ashes by -0.9‰ (MW test, *p* = 0.01; S6.8 Table in S6 Appendix) (Fig 3). This indicates that the presence of an ash layer covering foot elements have likely restricted the oxygen exchanges between bone apatite carbonates and the combustion atmosphere during and after the collapse of the pyre, or conversely, that the absence of such a layer have likely promoted oxygen exchanges between bone apatite carbonates and the atmospheric CO_2_ at the end of the cremation process. Charcoal generally presents lower δ^18^O values than the wood from which it originates (e.g. by about -9‰ for beech according to Schumacher et al. [94]). This suggests that below the ashes, a severely ^18^O-depleted atmosphere prevails, and that carbon and oxygen exchanges can still occur, which is not totally unsurprising as some of the ash layers were still hot (> 400°C) the next morning. The location below the ashes would therefore be a constraining condition that may promote the influence of the char-derived oxygen to the δ^18^O_carb_ signal. Interestingly, the final position of bones in ashes in addition to the presence of shoes and the initial position of the feet in the pyre seem to have a cumulative effect on the δ^18^O_carb_ values, with the greater difference in δ^18^O_carb_ values found between 1) the group of bones recovered above ashes but deriving from unshoed feet placed on top of the pyres, and 2) the group of bones recovered below ashes but deriving from shoed feet initially deposited at the bottom of the pyres (i.e. 3.7‰). Especially, this latter group contains the most negative δ^18^O_carb_ values from the whole dataset.

### The cyanamide content: The initial position in the pyre and the presence of footwear

The CN/P values derived from Agilent Technologies Cary 640 and Bruker Vertex 70v spectrometers are shown to be well correlated (y = 0.0944x – 0.0025; R^2^ = 0.88) (S7.1 Table in S7 Appendix). Due to its ability to work under vacuum, the Bruker Vertex 70v almost eliminates atmospheric disturbances, resulting in a decrease of the signal-to-noise ratio and thus in the measured cyanamide contents according to a ratio of about 13:1. A significance threshold of 0.02 for detecting the presence of cyanamide is proposed based on the instrument intercomparison carried out in this study and the work of Snoeck et al. [57].

The amount of cyanamide in calcined bone is relatively higher in shoed feet (mean = 0.042 ± 0.024, 1SD) than in unshoed ones (mean = 0.010 ± 0.010, 1SD) at the bottom of the pyre (pig 5) (MW test, *p* = 0.03; S6.9 Table in S6 Appendix), while a reverse pattern is observed between the shoed (mean = 0.010 ± 0.007, 1SD) and unshoed feet (mean = 0.044 ± 0.032, 1SD) located on top (pig 7) (MW test, *p* = 0.02; S6.9 Table in S6 Appendix) (S5.1 Table in S5 Appendix and see the other FTIR results for these feet in Section 4 in S1 Appendix). Overall, a slight top-to-bottom decrease in the mean ΔCN/P_shoed-unshoed_ values is observed (S5.2 Table in S5 Appendix). A joint effect of the initial position of the feet in the pyre and the presence of closed leather footwear seems to influence the CN/P values of the pig feet.

It is possible that leather contains low to moderate ammonia concentrations. Indeed, in leather processing, dehairing, deliming and/or softening are generally carried out with ammonium salts or urine derivatives, generating large amounts of ammonia [95–97]. The more reducing conditions surrounding the shoed feet, in conjunction with a higher presence of ammonia released during the combustion of the shoe, have likely promoted the incorporation of cyanamide into the apatite structure. Since the unshoed feet placed on the same deposition levels do not have cyanamide or have relatively low levels of cyanamide, it can be hypothesized that either the presence of ammonia in the combustion atmosphere was localized or reducing conditions were not present around the unshoed feet; one not excluding the other.

**Fig 6:**
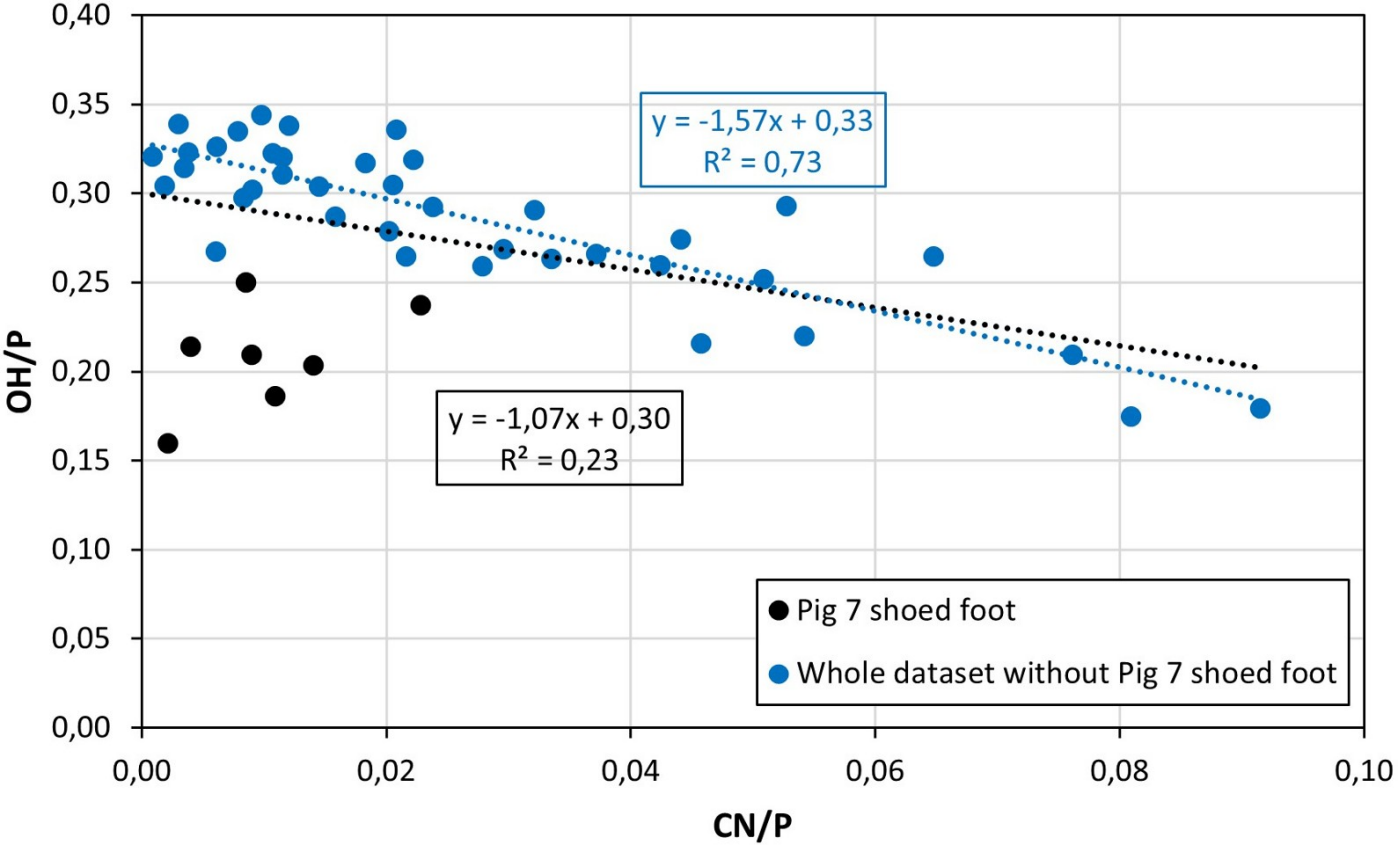
Biplot presenting the CN/P values against the OH/P values for pigs 5 to 8.

The OH/P and CN/P values of pigs 5 to 8 display a weak inverse correlation (R^2^ = 0.23) (Fig 6; S5.1 Table in S5 Appendix). This is caused by the shoed foot located on top of pyre 7. It presents the lowest mean OH/P value of the corpus (0.21), which likely is related to the collapse of pyre 7 and to the fall of the shoed foot out of the pyre. When this foot is excluded, the inverse correlation improves (R^2^ = 0.74) (Fig 6). Such a strong relationship confirms that cyanamide substitutes for hydroxyl groups within the bone matrices [57, 66]. However, there is no plausible explanation for the presence of cyanamide in the unshoed foot placed on top of pyre 7, although it is certainly related to the collapse of the structure and/or could be linked to the other inconsistent infrared indices obtained for this foot (see Section 4 in S1 Appendix and S5.1 Table in S5 Appendix). Furthermore, there is no statistically significant difference between the CN/P values of the feet deposited in the middle of pyres 6 and 8, with or without shoes (MW tests, *p* ≥ 0.11; S6.10 Table in S6 Appendix). Therefore, either the manure used in pyre 8 did not induce the expected ammonium-rich atmosphere or failed to produce cyanamide and its incorporation into the heated bones. Another explanation could be that the potentially reductive conditions necessary for the incorporation of cyanamide were not met at the time the manure was burned. Carrying out this experiment again but in bottom position should refine this interpretation. Finally, the position of bones in ashes does not influence the CN/P values (MW test, *p* = 0.99; S6.11 Table in S6 Appendix).

### Implications for archaeological cremations and future research

This study is a step forward to a more thorough comprehension of the cremation practices and rituals. The results obtained show that the initial positioning of feet on or within the pyre and the wearing of closed leather footwears may moderately-to-highly influence the oxygen isotope ratios of bone apatite carbonates and the cyanamide content of calcined bone. However, these effects should not be viewed as definitive or strict offsets, but rather as relative trends or orders of magnitude that can be expected when foot bones are cremated under similar conditions.

Because a multitude of cremation scenarios is technically possible, the number of parameters–controlled or not–coming into play is almost infinite. This is what makes the study of cremations challenging. However, previous work and ongoing research have shown that the oxygen isotope ratios of apatite carbonates in bone and the cyanamide content of calcined bone can vary locally (intra-site), regionally (inter-site), and chronologically (within and across time periods) [50, 54, 98]. The present study demonstrates that the initial positioning of feet, and thereby of the individual, as well as the wearing of closed leather shoes, and by extrapolation of clothing items, are plausible parameters that can explain these geographical and chronological variations. However, many other factors, that are not yet well circumscribed or understood, may also affect the oxygen isotope ratios of bone apatite carbonates and the cyanamide content of calcined bone. Among the extrinsic factors are all the actions of the living towards the body and the funeral pyre such as dressing and shoeing (with open or closed footwear) the deceased, tending and stirring the fire, reloading the fire with wood, placing offerings and grave goods. These extrinsic factors are influenced by practices and beliefs of the living and but also by the skills and know-how of the cremation operators (e.g. [35, 99]). Intrinsic factors include the type of materials used to build the pyre, the heating duration, the way fire is quenched, the body size of the deceased, the pyre settings, etc. All these parameters can theoretically lead to isotopic, structural and chemical changes similar to those observed. However, we believe that by sufficiently refining the sampling strategy and selecting the most relevant bones to answer specific archaeological questions, biomolecular archaeology can be of great interest and help. An important aspect of this work is that a single isotopic or infrared measurement per bone is likely to bias the interpretations that can be drawn from it. Although time consuming, only repetitive measurements per bone and per anatomical region studied can provide a reliable signal that can be exploited for archaeological purposes.

The replication of this study on forensic anthropological material is envisaged to definitively corroborate the results obtained on modern animals. It is very likely that the two feet of the same individual burned on an open-air pyre were treated in an identical manner, i.e. either shoed or unshoed. It can be hypothesized that foot bones enclosed in a closed leather shoe will have different isotopic and infrared values than leg bones from the same individual. Even if clothed, the leg will certainly not be in a garment as fire resistant as the leather of a shoe. The shield effect of any clothing relative to the leg will therefore be less than for a foot and this should be reflected in the results obtained. In addition, complementary data from well-defined archaeological contexts e.g. Pompeiian necropolises [20]) could help us better understand the observed variations and their potential meanings. Overall, this work and its multi-proxy approach / multi-sampling strategy represents a milestone with the potential to lead to a major breakthrough in cremation archaeology, and more broadly in archaeological science.

## Conclusion

This study demonstrates that from calcined foot bones it is possible to detect whether a deceased was wearing closed leather shoes during cremation and to identify the initial positioning of the feet, and thereby of the individual, on/in a pyre. By forming an additional protective layer around the foot, the footwear appears to temporarily delay the burning of the underlying pig tissues and, simultaneously, increase the heat-shielding effects of organic matter protecting the mineral fraction of bone. Bone apatite seems to be then exposed to fire at a more advanced stage of combustion of the pyre, during which carbonates exchange oxygen with a relatively more ^18^O-depleted atmosphere (due to the influence of lignin-derived oxygen). This results in more pronounced–and negative–shifts in the δ^18^O_carb_ values of shoed feet than feet burned bare (up to 2.5‰). Such an isotopic pattern is not recognized when the feet are placed on top of the pyre, probably due to the lack of physical constraint (pressure/weight of the logs above) to maintain the shoes closed, although weaknesses in the shoe patterns cannot be ruled out. Further research and experimental burnings need to be conducted to better understand the processes that occur on top of the pyres.

In addition, a concomitant isotopic effect of the initial location of the feet in the pyres is observed, resulting in a decrease in the δ^18^O_carb_ values of shoed feet by about 1.4‰ between each deposition level from the top to the bottom of the pyre. Another isotopic effect of approximately 0.9‰ is identified based on the relative position of the bones in relation to the ash heaps at the end of cremation. Bones recovered within the piles of ashes display lower values of δ^18^O_carb_ than bones recovered above the pile, probably due either to a decrease in oxygen intake into the bone apatite or to privileged oxygen exchanges with charcoal that are depleted in ^18^O. Finally, the presence of cyanamide (CN/P ≥ 0.02) is likely indicative of the wearing of unventilated footwear since the latter creates favorable conditions for its incorporation into the bones (reducing atmosphere and a greater amount of organic matter).

From an archaeological perspective, ritual sequences related to cremation practices are usually well identified, but generally poorly understood with regard to the organization of the burning act. This study opens new avenues to obtain additional information about the treatment of the deceased during cremation and the cremation operator’s range of skills. However, it is only when confronted with archaeological sources and field data that this approach yields its full potential. In the case of the presence of shoe nails in cremation-related deposits, it becomes possible to discuss if closed shoes were worn by the deceased or only placed next to the body on the pyre, which are different funerary gestures with possible different cultural or ritual meanings. Based on a multi-sampling strategy of the skeleton, and considering anthropological and anthracological data, it could be inferred if the pyre was left unattended during cremation or managed by operators. These are just a few examples of the promising prospects offered by isotopic and infrared evidence obtained from calcined bones.

## Supporting information

S1 Appendix Supporting text and figures for materials, methods, results and discussion sections. (DOCX)

S2 Appendix Heating temperatures in pyres. (XLSX)

S3 Appendix Isotopic results (δ^13^C_org_ and δ^15^N_org_) for unburnt leather and wood. (XLSX)

S4 Appendix Isotopic results (δ^13^C_org_, δ^15^N_org_, δ^13^C_carb_ and δ^18^O_carb_) for unburnt and burnt pig tissues (bone, muscle, skin and tendon). (XLSX)

S5 Appendix Infrared results for burnt pig bones (shoed and unshoed). (XLSX)

S6 Appendix Results from Mann-Whitney U tests. (XLSX)

S7 Appendix CN/P results from the instrument intercomparison. (XLSX)
