# Supplementary material for: These boots are made for burnin’: Inferring the position of the corpse and the presence of leather footwears during cremation through isotope (δ13C, δ18O) and infrared (FTIR) analyses of experimentally burnt skeletal remains
Source: PLoS One. 2021 Oct 13;16(10):e0257199. doi: 10.1371/journal.pone.0257199 (PMC8513878; doi:10.1371/journal.pone.0257199)
Supplement: S1 Appendix — (DOCX) [file pone.0257199.s001.docx]

These boots are made for burnin': Inferring the position of the corpse and the presence of leather footwears during cremation through isotope (δ^13^C, δ^18^O) and infrared (FTIR) analyses of experimentally burnt skeletal remains

Supporting Information

Kevin Salesse^1,2,3,4*^, Elisavet Stamataki^1,3,4^, Ioannis Kontopoulos^5^, Georges Verly^6^, Rica Annaert^3,7^, Mathieu Boudin^8^, Giacomo Capuzzo^1^, Philippe Claeys^4^, Sarah Dalle^3,9^, Marta Hlad^1,3,4^, Guy de Mulder^9^, Charlotte Sabaux^1,9^, Amanda Sengeløv^1,9^, Barbara Veselka^3,4^, Eugène Warmenbol^10^, Martine Vercauteren^1^, Christophe Snoeck^3,4,11^

^1^Research Unit: Anthropology and Human Genetics, Department of Biology of Organisms and Ecology, Université Libre de Bruxelles, CP192, Avenue F.D. Roosevelt 50, 1050 Brussels, Belgium.

^2^UMR 5199: “PACEA - De la Préhistoire à l'Actuel: Culture, Environnement et Anthropologie”, University of Bordeaux, Building B8, Allée Geoffroy St. Hilaire, CS 50023, 33615 Pessac cedex, France.

^3^Maritime Cultures Research Institute, Department of Art Sciences and Archaeology, Vrije Universiteit Brussel, Pleinlaan 2, 1050 Brussels, Belgium.

^4^Research Unit: Analytical, Environmental and Geo-Chemistry, Vrije Universiteit Brussel, AMGC-WE-VUB, Pleinlaan 2, 1050, Brussels, Belgium.

^5^University of Copenhagen, GLOBE Institute, Section for GeoGenetics, Øster Voldgade 5-7, 1350 København, Denmark.

^6^Sorbonne Université, Faculté des Lettres, 1, rue Victor Cousin, 75005 Paris, France.

^7^Flemish Heritage Agency, Havenlaan 88/5, 1000 Brussels, Belgium

^8^Royal Institute for Cultural Heritage, Jubelpark 1, 1000 Brussels, Belgium.

^9^Department of Archaeology, Ghent University, Sint-Pietersnieuwstraat 35, 9000 Ghent, Belgium.

^10^Centre de Recherches en Archéologie et Patrimoine, Department of History, Arts, and Archaeology, Université Libre de Bruxelles, CP133, Avenue F.D. Roosevelt 50, 1050 Brussels, Belgium.

^11^G-Time Laboratory, Université Libre de Bruxelles, CP160/02, Avenue F.D. Roosevelt 50, 1050 Brussels, Belgium.

* Corresponding author

E-mail: Kevin.Salesse@vub.be (KS)

1. **Supporting Information for Materials**

*Pyre architecture:* Each pyre was made of eight levels of firewood pieces (≃ 300 kg of wood) arranged in stacked rows (Fig S1.1). The longer pieces (100 x 15 cm) were used to construct the four lowest levels whereas the shorter ones (80 x 15 cm) were used to build the upper part of the pyre (Fig S1.1; see movie at https://www.youtube.com/watch?v=iC2wVjcjG_E&t=1s). The wood pieces were placed to ensure maximal stability to the structure. Particular attention was paid to minimize the size of interstices between logs to reduce the incoming airflow in the pyre and increase combustion time.


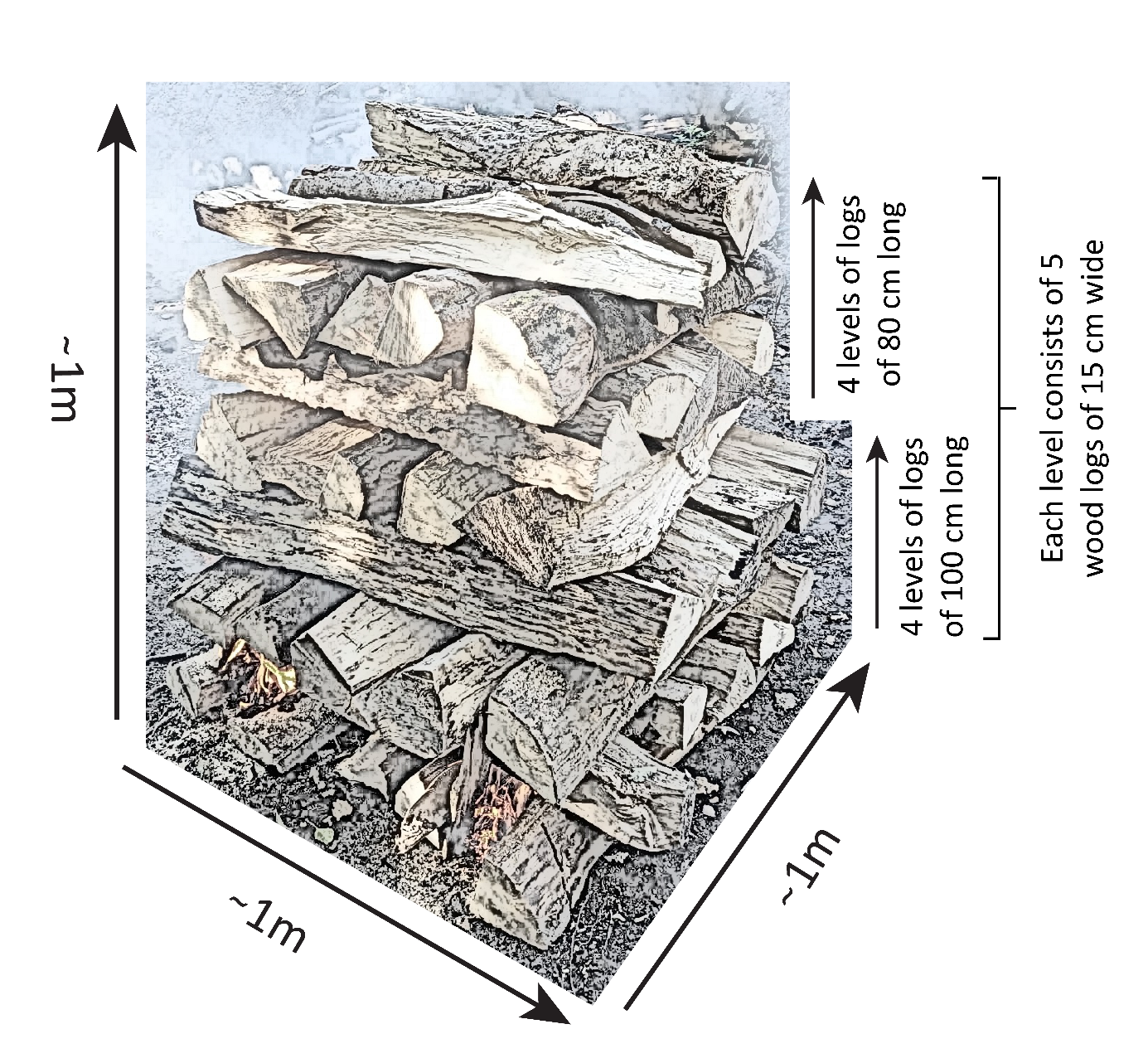


**Fig S1.1. Sketch of a pyre built for the outdoor burning experiments.**

*Leather manufacturing process*: The purchased animal hides – the neck part of a cowhide (14 ft²) and a whole goatskin (8 ft²) – were subjected to a limited number of chemical treatments. Briefly, a soaking, wetting and degreasing agent were used to wash the skins while lime, sodium sulfide and enzymatic products were used to dehair them and remove the inter-fibrillary matter and non-structural proteins. Sulfuric acid and salt containing ammonium were employed to pickle the hides. Vegetable tannins were used for tanning and a tallow-paraffin mixture was applied to soften the leather hides and make them waterproof.

*Footwear making:* To create the shoe, a small leather cut (30 x 27 cm) was folded in half along the longer side and laced on the three remaining sides (pre-perforated every 2.5 cm; Ø 3 mm) with leather strips made from the same hide (Fig S1.2; see movie at https://www.youtube.com/watch?v=Pj612ShvowQ). In the first session (October 2018), three of the shoes used were made of cow leather and one of goat leather, whereas in the second session (July 2019), all shoes were produced using cow leather.


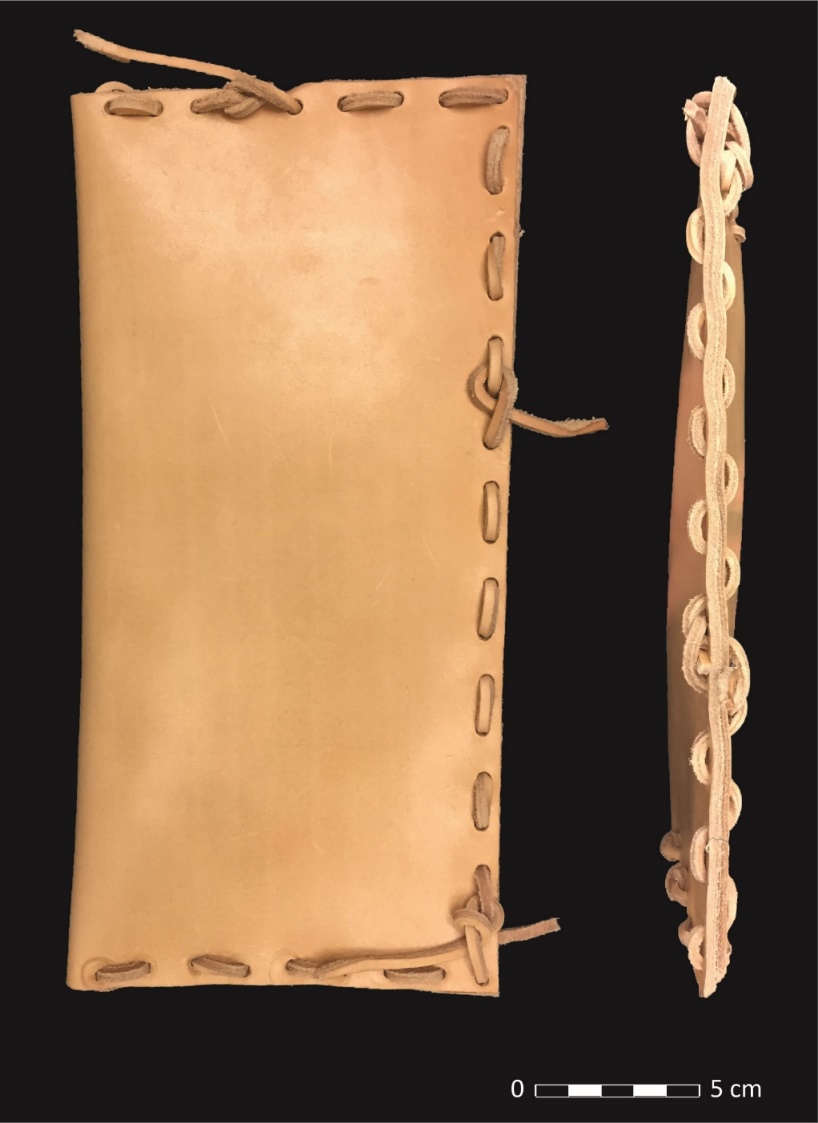


**Fig S1.2. Example of a lace-up shoe designed for the experimental cremations.**

1. **Supporting Information for Methods**

*Defatting procedure based on Kates [1] and Liden et al. [2]:* The powdered bone samples were soaked in a methanol-chloroform mixture (2:1, v/v) and placed in an ultrasonic bath. After 20 to 30 min, the supernatant containing lipids was discarded and the solution was renewed. This step was repeated at least three times until the fatty acids and their derivatives were completely eliminated. The samples were thoroughly rinsed and oven-dried at 50°C overnight.

*Collagen extraction protocol based on Brock et al. [3]:* Bone powder samples (amount: 100 to 400 mg) were demineralized with 0.5 M hydrochloric acid (HCl) at 4°C for 48 h. They were placed in 0.1 M sodium hydroxide (NaOH) at room temperature for 30 min to remove organic acids and rinced with 0.5 M HCl at room temperature for 15 min. The samples were washed several times with ultrapure (MilliQ^TM^) water between each reagent. Gelatins were subsequently solubilized in 0.01M HCl at 70°C for 48 h and filtered using a 60-90-µm polyethylene Ezee-filters^TM^ to trap possible impurities. Collagen samples were freeze-dried at -54°C for 48 h.

*Pre-treatment of unburnt bone carbonate samples according to Balasse et al. [4]:* Bone powder samples (amount: ≈ 35 mg) were treated with 2-3% sodium hypochlorite (NaClO) at room temperature for 72 h to remove organic matter, and then with 2 mL of 0.1 M acetic acid (CH_3_COOH) at room temperature for 4 h to remove hypothetical exogenous carbonates. NaClO and CH_3_COOH solutions were refreshed every 24 h and half of the time, respectively. Samples were rinsed three times with MilliQ^TM^ water between the two steps and at the end of the procedure and finally oven-dried at 50°C overnight.

Even though it can influence the carbon and oxygen isotope composition of bone apatite carbonates (see Pellegrini & Snoeck 2016), the use of NaClO was deemed necessary as modern bone contains large amounts of organic material that could impair the correct reaction between bone apatite carbonates and phosphoric acid used during the measurement by IRMS. Furthermore, the differences investigated here between unburnt and burnt bone are much larger than any potential bias that could be induced by different pre-treatments.

*Pre-treatment of burnt bone carbonate samples according to Snoeck et al. [5]*: Bone pieces (amount: 100 to 200 mg) were rinsed three times with MilliQ^TM^ water for 10 min, and treated with 10 mL of 1 M CH_3_COOH at room temperature for 3 to 10 min and washed three times with MilliQ^TM^ water for 10 min to eliminate pyre- and soil-derived contaminants. Each step was performed in an ultrasonic bath. The samples were left overnight in an oven at 50°C. Once dry, the samples were crushed into a powder using a mortar and pestle.

*Sample preparation for FTIR analysis based on Kontopoulos et al. [6]:* The inner and outer surfaces of the bone fragments were cleaned using a scalpel. Bone samples (amount: 50 to 100 mg) were crushed using a pestle and mortar and sieved using two woven stainless-steel mesh sieves to retain powder with a particle size of 25-50 μm.

*Measured FTIR indices:* The infrared splitting factor (IRSF), which provides information about bone apatite crystallinity (crystal size and structural order/disorder), was calculated as described by Weiner and Bar-Yosef [7]: the sum of the absorbances of the two ν_4_PO_4_ bands at 565 and 605 cm^-1^ was divided by the absorbance of the valley at 595 cm^-1^. The carbonate-to-phosphate ratio (C/P), used to assess the carbonate content in bone apatite relative to the phosphate content, was determined according to Wright and Schwarcz [8]: the intensity of the ν_3_CO_3_ band at 1415 cm^-1^ was divided by that of the ν_3_PO_4_ band at 1035cm^-1^. The amide-to-phosphate ratio (Am/P), which indicates the amount of organic matter still present in heated bones relative to the phosphate content, was assessed after Trueman et al. [9]: the latter is measured by the intensity ratio of the ν_1_Amide band at 1640 cm^-1^ over the one of the ν_3_PO_4_ band at 1035 cm^-1^. The carbonyl-to-carbonate ratio (C/C) is temperature-dependent and was defined by Thompson et al. [10]: it compares two absorbance wavelengths associated to carbonate, i.e. the ν_3_CO_3_ band (carbonates A and B) at 1450 cm^-1^ versus the ν_3_CO_3_ band (carbonate B) at 1415 cm^-1^. The type B carbonate-to-phosphate index (BPI), which provides indication of the amount of carbonate B, was determined following Sponheimer and Lee-Thorp [11]: i.e. the ν_3_CO_3_ band at 1415 cm^-1^ over the ν_4_PO_4_ band at 605 cm^-1^. The hydroxyl group-to-phosphate ratio (OH/P), which describes the changes to hydroxyl groups in heated bone apatite, was measured according to Snoeck et al. [12]: the intensity of the OH band at 630 cm^-1^ is divided by the one of the ν_4_PO_4_ band at band at 605 cm^-1^. The cyanamide-to-phosphate index (CN/P), which reveals the relative amount of cyanamide in calcined remains, was estimated by absorbance of the CN_2_H band at 2010 cm^‑1^ ratioed to the ν_4_PO_4_ band at band at 605 cm^-1^ based on Zazzo et al. [13] and Snoeck et al. [12].

*Statistics:* Statistical analyses were performed using the free-to-use software PAST 3 (<https://www.nhm.uio.no/english/research/infrastructure/past/>). The nonparametric Mann-Whitney U (MW) test was used to test whether two independent groups of isotopic or infrared data were likely to derive from the same population.

1. **Supporting Information for Results**

*Isotopic variability of the leathers:* The cowhide yields a mean δ^13^C_org_ value of -28.3 ± 0.2‰ (1SD) while the goatskin has a mean δ^13^C_org_ value of -27.3 ± 0.2‰ (1SD) (Table S3.1 in S3 Appendix). Cowhide and goatskin have significantly different δ^13^C_org_ values (Mann-Whitney (MW) test, *p* < 0.01; Table S6.12 in S6 Appendix). There is no significant difference in δ^13^C values between the top, inner and bottom parts of each hide (also called full-grain, top-grain and genuine leathers, respectively) (MW tests, *p* ≥ 0.10; Table S6.13 in S6 Appendix). This suggests that the paraffin has a similar δ^13^C signature to that of the cowhides and the goatskin and/or is used in such a small quantity that it does not alter the carbon isotope composition of the whole hides.

*Isotopic variability of the wood:* Beech wood used to build the pyres has δ^13^C_org_ values ranging from -30.9 and -23.7‰ (mean = -27.6 ± 1.6‰, 1SD, n = 53) (Table S3.2 in S3 Appendix). Although these values do not statistically significantly differ from those of the hides/skins (MW test, p = 0.51; Table S6.1 in S6 Appendix) (Fig 2), the firewood induces a greater depletion in ^13^C of the combustion-derived CO_2_ pool, which ultimately results in a larger shift in the δ^13^C values of calcined bones from the outdoor burning experiments compared to the laboratory experiments.

1. **Supporting Information for Discussion**

*Chemical and structural changes in calcined bones:* The infrared indices show that the remains from pigs 5 to 7 were subjected to moderate-to-high intensity burnings (Table S5.1 in S5 Appendix), which is consistent with our records in the field (Table S2.1 in S2 Appendix). The Am/P values are extremely low (maximum value of 0.01) confirming a complete removal of the organic matter from all samples, as expected in calcined bone. Moreover, the relatively low C/P and BPI values, ranging from 0.02 to 0.07 (mean = 0.05 ± 0.01, 1SD) and from 0.04 to 0.14 (mean = 0.07 ± 0.02, 1SD), and the relatively high C/C values, ranging from 1.08 to 1.68 (mean = 1.36 ± 0.12, 1SD), indicate that carbonates in the bone matrices decreased in significant amounts during the heating process. High crystallinity was observed, with IRSF values ranging from 4.38 to 6.06 (mean = 4.99 ± 0.45, 1SD), which is characteristic of fully calcined bones.

The feet placed at the bottom of the pyre differ from those deposited in the middle according to the IRSF and C/C values (Mann-Whitney tests; *p* ≤ 0.03; Table S6.14 in S6 Appendix), while the OH/P data discriminate the feet placed on the top of the pyre from those deposited in the middle or at the bottom (MW tests; *p* ≤ 0.02; Table S6.14 in S6 Appendix). The unshoed and shoed feet differ from each other according to the IRSF, C/P and OH/P data are similar (MW tests, *p* ≤ 0.01; Table S6.15 in S6 Appendix), whereas the C/C and BPI values (MW tests, p ≥ 0.21; Table S6.15 in S6 Appendix). All this tends to suggest that shoed feet were exposed to slightly higher temperatures than the unshoed feet. The temperature heterogeneity could be simply circumstantial or an effect of the presence of the shoe leather. The addition of organic matter around the feet could have allowed the foot bones to reach higher temperatures, although more data are needed to test this hypothesis.

The inter-pyre comparisons reveal no specific trends based on the infrared indices. This may be inherent in the limited amount of data available and pigs tested. Finally, bones recovered above or within the piles of ashes exhibit similar values for the different infrared indices tested (MW tests; p ≥ 0.18 Table S6.16 in S6 Appendix). The relative position of the bones in relation to the ash heaps at the end of cremation does not seem to influence the chemical and structural characteristics of calcined bones.

With regard to the specific case of pyre 7 that collapsed and from which the shoed foot was expelled, inconsistent patterns in the infrared indices are observed. While the BPI and C/P values for the unhsoed foot are aligned with the same linear relationship as the rest of the corpus, the BPI and C/P values for the shoed foot deviate significantly (Fig S1.3). Moreover, the shoed foot exhibits the lowest OH/P values of this study (Fig 8). Compared to the unshoed foot bones, the shoed ones would have lost relatively less carbonates and been subjected to less replacements of A-type carbonates by hydroxyl groups. However, it cannot be completely ruled out that once the footwear fell onto the ground, it would have incorporated sediment, such as sand, which would have ultimately affected the infrared indices as described above.

**Fig S1.3. Biplot presenting the BPI values against the C/P ratios for pigs 5 to 8.**

**References**

1. Kates M. Techniques of lipidology: analysis and identification of lipids. Amsterdam: Elsevier; 1986. 464 p.

2. Liden K, Takahashi C, Nelson DE. The effects of lipids in stable carbon isotope analysis and the effects of NaOH treatment on the composition of extracted bone collagen. Journal of Archaeological Science. 1995;22(2):321-6. doi: http://dx.doi.org/10.1006/jasc.1995.0034.

3. Brock F, Geoghegan V, Thomas B, Jurkschat K, Higham TFG. Analysis of Bone “Collagen” Extraction Products for Radiocarbon Dating. Radiocarbon. 2013;55(2):445-63. doi: 10.1017/S0033822200057581.

4. Balasse M, Ambrose SH, Smith AB, Price TD. The seasonal mobility model for prehistoric herders in the south-western Cape of South Africa assessed by isotopic analysis of sheep tooth enamel. Journal of Archaeological Science. 2002;29(9):917-32.

5. Snoeck C, Lee-Thorp J, Schulting R, de Jong J, Debouge W, Mattielli N. Calcined bone provides a reliable substrate for strontium isotope ratios as shown by an enrichment experiment. Rapid Communications in Mass Spectrometry. 2015;29(1):107-14. doi: 10.1002/rcm.7078.

6. Kontopoulos I, Presslee S, Penkman K, Collins MJ. Preparation of bone powder for FTIR-ATR analysis: The particle size effect. Vibrational Spectroscopy. 2018;99:167-77. doi: https://doi.org/10.1016/j.vibspec.2018.09.004.

7. Weiner S, Bar-Yosef O. States of preservation of bones from prehistoric sites in the Near East: A survey. Journal of Archaeological Science. 1990;17(2):187-96. doi: http://dx.doi.org/10.1016/0305-4403(90)90058-D.

8. Wright LE, Schwarcz HP. Infrared and isotopic evidence for diagenesis of bone apatite at Dos Pilas, Guatemala: palaeodietary implications. Journal of Archaeological Science. 1996;23(6):933-44. doi: 10.1006/jasc.1996.0087. PubMed PMID: WOS:A1996VQ57200013.

9. Trueman CNG, Behrensmeyer AK, Tuross N, Weiner S. Mineralogical and compositional changes in bones exposed on soil surfaces in Amboseli National Park, Kenya: Diagenetic mechanisms and the role of sediment pore fluids. Journal of Archaeological Science. 2004;31(6):721-39. doi: http://dx.doi.org/10.1016/j.jas.2003.11.003.

10. Thompson TJU, Gauthier M, Islam M. The application of a new method of Fourier Transform Infrared Spectroscopy to the analysis of burned bone. Journal of Archaeological Science. 2009;36(3):910-4. doi: https://doi.org/10.1016/j.jas.2008.11.013.

11. Sponheimer M, Lee-Thorp JA. Alteration of enamel carbonate environments during fossilization. Journal of Archaeological Science. 1999;26(2):143-50.

12. Snoeck C, Lee-Thorp JA, Schulting RJ. From bone to ash: Compositional and structural changes in burned modern and archaeological bone. Palaeogeography, Palaeoclimatology, Palaeoecology. 2014;416:55-68. doi: https://doi.org/10.1016/j.palaeo.2014.08.002.

13. Zazzo A, Lebon M, Chiotti L, Comby C, Delqué-Količ E, Nespoulet R, et al. Can we Use Calcined Bones for 14C Dating the Paleolithic? Radiocarbon. 2013;55(3):1409-21. Epub 02/09. doi: 10.1017/S0033822200048347.
